# Changes of cytokine levels in a mouse model of post-infectious irritable bowel syndrome

**DOI:** 10.1186/s12876-015-0272-8

**Published:** 2015-04-01

**Authors:** Bo Yang, Xuchun Zhou, Cheng Lan

**Affiliations:** 1 Department of Gastroenterology, The First Affiliated Hospital of Chongqing Medical University, Chongqing, 4000 l6 China; 2 Department of Gastroenterology, Hainan Provincial People’s Hospital, Haikou, 570311 China

**Keywords:** Post-infectious irritable bowel syndrome, IL-1β, IFN-γ, IL-10, IL-17

## Abstract

**Background:**

Irritable bowel syndrome (IBS) is a highly prevalent functional gastrointestinal disorder. Post-infectious IBS (PI-IBS) is caused by an acute gastrointestinal infection preceding the onset of symptoms. However, the pathophysiology of PI-IBS is not clear, and the purpose of this study was to investigate the probable immune mechanisms of PI-IBS.

**Methods:**

C57BL/6 mice were randomly assigned to either an infection group or a control group. Mice in the infection group were infected with *Trichinella spiralis* to establish a model of PI-IBS (500 Trichinella), while control mice received only salt solution. Visceral sensitivity of colorectal distention in mice was evaluated by abdominal withdrawal reflex scores and intestinal inflammation was assessed using hematoxylin-eosin staining; at day 56 post-infection, the mRNA and protein levels of specific cytokines in the gut segments were detected using reverse-transcription polymerase chain reaction and enzyme-linked immunoabsorbent assay.

**Results:**

Levels of interferon γ and interleukin (IL)-17 in the PI-IBS group were significantly increased in the duodenum and ileum, and IL-10 was decreased in the jejunum, ileum, and colon compared with control mice. However, the expression level of IL-1β was not significantly different between the two groups.

**Conclusions:**

The present study suggests that the local low-grade inflammation and immune activation that are an important component of the pathophysiology of PI-IBS are primarily induced and maintained by specific cytokines.

## Background

Irritable bowel syndrome (IBS) is a highly prevalent functional gastrointestinal disorder characterized by abdominal pain and alterations in bowel habits [1]. Between 3.7% and 36% of patients with acute gastrointestinal infection subsequently develop a form of IBS known as post-infectious IBS (PI-IBS) [2].

Many pathogens can cause PI-IBS. Bacteria and parasites are often used in PI-IBS animal models; however, for bacterially-induced PI-IBS animal models, the major characteristics of IBS such as visceral hypersensitivity, alterations in motility and secretion are weak or sometimes absent, and it therefore remains controversial whether bacterial infection results in a valid model of PI-IBS. *Nippostrongylus brasiliensis* and *Cryptosporidium parvum* have been used in rat models of PI-IBS. However, it was found that models infected by *N. brasiliensis* lacked visceral sensitivity. The features of IBS such as motility dysfunction and altered secretion have not been evaluated in the *C. parvum* infection model [3]. Infection by *Trichinella spiralis* larvae induced changes in visceral sensitivity, alterations of intestinal smooth muscle function, and altered secretion. These abnormalities persisted after animals recovered from infection, suggesting that this is a suitable model of PI-IBS [4,5].

The pathophysiology of PI-IBS is not fully understood, but low-grade inflammation and chronic alteration of the immune system at the molecular level have been shown to be associated with mucosal secretory function, and with smooth muscle and enteric nervous fibers [6-8]. In particular, an imbalance of pro- and anti-inflammatory cytokines is seen, which may play a key role in the local intestinal inflammation.

A number of reports related to PI-IBS patients have found a clear increase in many immune cell types within the mucosa [9-15]. Various animal models have been developed to gain insight into the underlying mechanism of IBS. A large number of studies have demonstrated that certain indicators, such as visceral hypersensitivity and persistent dysfunction of the intestinal muscle, exist in mice infected with *T. spiralis* [4,16,17]. In fact, the inflammatory response to intestinal parasites has been regarded as a representative defense response against pathogens. For this reason, experimental infection with the parasite *Trichinella* has been widely used to establish models for detecting the pathogenesis of intestinal dysfunction [18,19]. Activated immune cells continue to release various cytokines after an acute intestinal infection [20], for example, T-helper (Th) cells produce interferon (IFN)-γ and interleukin (IL)-1β to promote the inflammatory response; T-regulatory cells release IL-10 to prevent autoimmunity; in contrast, IL-17, which is produced by Th17 cells, can induce autoimmunity [20]. These cytokines may alter the physiology and immunity of the host gut to cause symptoms of PI-IBS: IL-1β activates nitric oxide synthase and enkephalin immunoreactivity on interneurons or motorneurons and suppresses presynaptic cholinergic neurotransmission [21,22]. Moreover, IL-1β can stimulate cecocolonic motor activity and cause a migrating myoelectric complex pattern in the small intestine [23]. IFN-γ and IL-1β can disrupt the colonic epithelial barrier and increase intestinal tight junction permeability [24-26]; reduction of IL-10 may cause an imbalance between pro- and anti-inflammatory mechanisms, resulting in chronic intestinal inflammation [27]. IL-17 and IFN-γ can act cooperatively in the promotion of inflammatory responses in the intestinal mucosa [28]. Hence, in this study, we established a PI-IBS mouse model and assessed local expression levels of a range of cytokines in different intestinal segments in order to investigate the probable immune mechanisms of PI-IBS.

## Methods

### Animals

Studies were performed on specific pathogen-free female C57L/B6 mice, 4–6 weeks old, obtained from the Animal Center of Chongqing Medical University. The experimental procedure was approved by the Animal Welfare committee of Chongqing Medical University, China. A total of 34 mice were randomly assigned to either a control group (n = 17) or a PI-IBS group (n = 17) and maintained under controlled conditions with 12-h light/dark cycle.

### *Trichinella* infection

Infective larvae were obtained from muscle of C57L/B6 mice infected with *Trichinella* at least 30 days in advance. The infected mice were humanely sacrificed, skinned, and eviscerated, and the muscles containing encysted larvae were minced and digested in 1% pepsin A (Biosharp, China) and 1% HCl at 37°C for 16 hours. The isolated infective larvae were washed several times with 0.85% NaCl and suspended in balanced salt solution. Mice in the PI-IBS group were infected by the oral administration of 350–400 larvae in 0.2 ml of solution, while mice in the control group received the same volume of physiological saline [11].

### Sample collection and processing

Three mice from each group were humanely sacrificed (ether inhalation and cervical dislocation) on day 14, 28 and 56 post-infection (PI). Intestinal samples taken from the duodenum (10 cm distal to the pylorus), jejunum (20 cm distal to the pylorus), ileum (30 cm distal to the pylorus) and colon (distal to the caecum) were flushed with physiological saline to remove gut contents. A 1 cm long sample from each intestinal tissue was fixed overnight in 4% paraformaldehyde and embedded in paraffin for histological analysis. The rest of the intestine tissue was immediately preserved in liquid nitrogen for subsequent RNA extraction and protein assay.

### AWR scores

Visceral hyperalgesia to colorectal distention was assessed at day 56 PI by abdominal withdrawal reflex (AWR) [29]. Mice were briefly anesthetized with ether, and a balloon catheter (6-Fr, 2 mm external diameter) was inserted rectally into the descending colon of mildly sedated mice. After waking up and adapting for 1 h, colorectal distention was performed in a stepwise fashion. Each 20-second distention was followed by a 30-second resting period. Each level of distention (0.25, 0.35, 0.5, and 0.65 ml) was repeated three times, and the balloon was deflated and withdrawn after assessing AWR. The AWR score was assigned as follows: 0, no behavioral response to colorectal distention; 1, brief head movement followed by immobility; 2, contraction of abdominal muscles; 3, lifting of abdomen; 4, body arching and lifting of pelvic structures [30].

### Histological analysis

Paraffin-embedded tissues were cut into 5-μm-thick sections. To deparaffinize, the sections were immersed in xylene at 56°C twice for 20 min, and hydrated with ethanol (twice with 100%, once with 95%, and once with 75% ethanol) for 5 min. The sections collected at the selected time points (14, 28 and 56 days PI in two mice from each group), were processed routinely for hematoxylin and eosin (H&E) histology. H&E-stained slides were evaluated in a blinded fashion by two independent investigators. A histopathological score was assigned as described by Dieleman [31].

### RT-PCR mRNA assay

Total RNA from the intestinal mucosa was extracted using Trizol solution (Takara, Japan). The expression of cytokine genes was assayed using reverse-transcriptase polymerase chain reaction (RT-PCR). The β-actin (*Actb*) mRNA level was used as an internal reference, and levels of mRNA expression were quantitated by optical densitometry after electrophoresis on an agarose gel. All primers are listed in Table 1. The reverse transcription was conducted at 37°C for 15 min, 95°C 5 sec. The PCR cycling condition was 36 cycles at 94°C for 40 sec, 55°C (*Il10* and *Il1b*), 57°C (*Il17* and *Ifng*) or 59°C (*Actb*) for 30 sec and 72°C for 35 sec. The PCR end products were run on a 5% agarose gel and stained with ethidium bromide. The gray values of the bands were calculated using quantity one software (Bio-Rad, America). The relative mRNA expression levels of the target genes were normalized to the corresponding internal standard.

**Table 1:** **RT-PCR primer sequences**

Cytokines	Primer sequence
*Il1b* (94 bp)	Forward 5′—ATGGGCAACCACTTACCTATTT—3′
	Reverse 5′—GTTCTAGAGAGTGCTGCCTAATG—3′
*Il10* (116 bp)	Forward 5′—ACAGCCGGGAAGACAATAAC—3′
	Reverse 5′—CAGCTGGTCCTTTGTTTGAAAG—3′
*Il17* (123 bp)	Forward 5′—CGCAATGAAGACCCTGATAGAT—3′
	Reverse 5′—CTCTTGCTGGATGAGAACAGAA—3′
*Ifng* (128 bp)	Forward 5′—AAATCCTGCAGAGCCAGATTAT—3′
	Reverse 5′—GCTGTTGCTGAAGAAGGTAGTA—3′
*Actb* (470 bp)	Forward 5′—AGGCTGTGCTGTCCCTGTATG—3′
	Reverse 5′—GAGGTCTTTACGGATGTCAACG—3′

### Intestine homogenate and ELISA

For enzyme-linked immunoabsorbent assay (ELISA) [32], all of the intestinal segments were processed using an ultrasonic disrupter (Bandelin, Germany) and homogenized in RIPA buffer (Takara, Japan). The homogenates were then centrifuged at 10000 rpm for 20 min, and protein concentrations in the supernatant of homogenates were determined using a BCA protein assay kit (Beyotime, China). Levels of tissue cytokines were assayed using mouse IL-1β, IL-10, IL-17, and IFN-γ ELISA kits (Boster, China) according to the manufacturer’s protocols.

### Statistical analysis

Statistical analysis was performed using SPSS 19.0 software. Values are presented as mean ± standard deviation (SD). The independent sample t test was used to compare the results between the two groups. A value of *P* < 0.05 was accepted as statistically significant.

## Results

### AWR scores

AWR scores in the PI-IBS group were significantly higher than those in the control group at distention volumes of 0.35 and 0.5 ml (*P <* 0.01). However, there were no significant differences at volumes of 0.25 or 0.65 ml (*P* > 0.05). This was because distention at a volume of 0.25 ml was too slight to reach the minimum threshold pressure of abdominal muscle contraction, whereas distention at a volume of 0.65 ml was so strong that it led to a very intense response in mice of both groups (Table 2).

**Table 2:** **AWR scores for colon distention in mice in the PI-IBS and control groups** $\left(\overline{\mathbf{x}}\pm \mathbf{s}\right)$

Groups	n	Volume of colon distention			
		0.25 ml	0.35 ml	0.5 ml	0.65 ml
PI-IBS	8	0.50 ± 0.25	2.42 ± 0.24**	3.54 ± 0.31**	3.88 ± 0.35
Control	8	0.33 ± 0.31	1.83 ± 0.44	2.92 ± 0.43	3.75 ± 0.46

All data are presented as the mean ± SD. **P < 0.01 compared with the control group.

### Histological analysis

**Figure 1:**
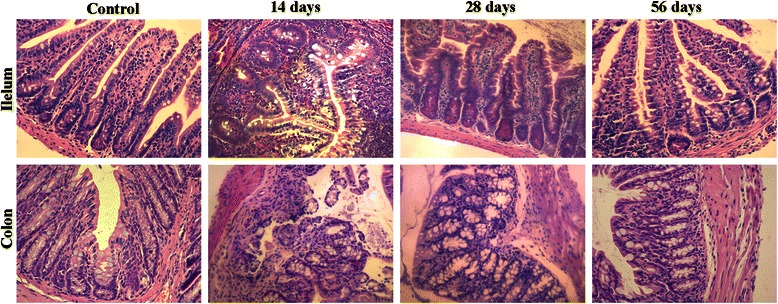
**Histological analysis of intestinal tissue samples.** Sections of ileum and colon from control or infected (at 14, 28, or 56 days PI, n = 3) mice were stained with hematoxylin and eosin (H&E). Original magnification × 200.

H&E staining of the ileum and colon showed a marked infiltration by neutrophils in the lamina propria and interstitial edema on day 14 PI. Infiltration and edema gradually reduced from day 14 PI until day 56 PI, at which stage no obvious inflammatory infiltrate was observed (Figure 1).

### ELISA

**Figure 2:**
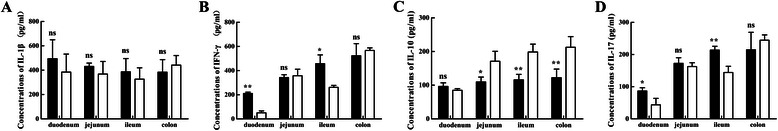
**Concentrations of IL-1β (A), IL-10 (B), IL-17 (C), and IFN-γ (D) protein in the different intestinal segments in the PI-IBS (n = 8) and control (n = 8) groups.** Black: PI-IBS group, white: control group. Data are presented as mean ± SD. *P < 0.05, **P < 0.01 versus control group.

The localized expression of IL-1β, IL-10, IL-17, and IFN-γ protein in the intestine was assayed using the corresponding ELISA kits. As shown in Figure 2, expression of IL-10 in jejunum, ileum and colon was much lower in the PI-IBS group than that in control group (all *P* < 0.05). In contrast, compared with control mice, the concentrations of IL-17 and IFN-γ in the duodenum and ileum of PI-IBS mice were significantly higher (all *P* < 0.05). However, the concentration of IL-1β showed no noticeable differences in any of the intestinal segments between the two treatment groups.

### RT-PCR mRNA assay

**Figure 3:**
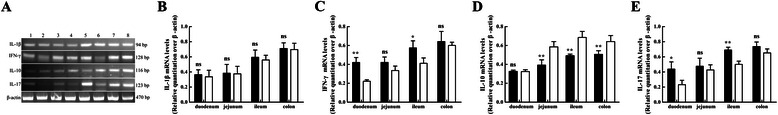
**The mRNA levels of*Il1b*(B),*Il10*(C),*Il17*(D), and*Ifng*(E) in different intestinal segments in the PI-IBS (n = 8) and control (n = 8) groups. A**. Representative graphs of the PCR assay. Lanes 1, 3, 5, and 7 are duodenum, jejunum, ileum and colon of the PI-IBS group, respectively; lanes 2, 4, 6, and 8 are duodenum, jejunum, ileum and colon of the control group, respectively. Black: PI-IBS group, white: control group. Date are presented as mean ± SD. *P < 0.05, **P < 0.01 versus control group.

*Il10* levels in the jejunum, ileum, and colon segments were significantly lower in the PI-IBS group than those in control group (all *P* < 0.05). For *Il17* and *Ifng* levels, those in the duodenum and ileum were notably higher in PI-IBS mice compared with control mice (all *P* < 0.05). However, the *Il1b* mRNA levels were not significantly different between the two groups (Figure 3).

## Discussion

Eight weeks after *T. spiralis* infection, the GI system still had disturbed visceral hypersensitivity without any histological evidence of intestinal inflammation. This means that the *T. spiralis* infection model is an acceptable model to represent PI-IBS [33]. AWR scores were altered in response to low or medium pressures, but did not significantly differ from healthy mice at high pressure. This indicates that the low/medium threshold nerves, but not the high threshold nerves, are altered. This may be related to different mechanical stimulation activated by different pressure expansion [34]. When distention volume was 0.25 ml, the pressure was too low to cause any visceral sensation. When the distention volume was 0.65 ml (high pressure), the level of stimulation was so high that it resulted in a very intense response in both groups of mice. When the distention volume was either 0.35 or 0.5 ml, the AWR scores in the model group were higher than those in the control group, and the pain threshold in the model group was lower than that in the control group at the same time point. This suggests an increase of the visceral sensitivity in mice after infection. Although the pathogenesis of PI-IBS is not well understood, increasing evidence suggests that low-grade inflammation and immune activation play a pivotal role in the occurrence and persistence of its symptoms [35-40]. Several reports have described high numbers of T cells in various lymphoid compartments of the small or large intestine in IBS patients [9,10,41], and activated T cells produce many cytokines involved in the inflammatory process, including IL-1β, IL-10, IL-17, and IFN-γ. Further studies have shown that chronic alterations of inflammatory cytokines are found in the peripheral blood and intestinal mucosa, which is consistent with the development of IBS symptoms at the molecular level [20,37]. However, alterations of certain cytokines in different locations within the GI tract have not been systematically reported in PI-IBS.

In the present study, we successfully established a PI-IBS mouse model induced by *Trichinella* larvae and found that the levels of IFN-γ, as well as IL-17, were increased in the duodenum and ileum. IFN-γ is a classical pro-inflammatory cytokine, which can act cooperatively with IL-17 in the promotion and shaping of inflammatory responses in the intestinal mucosa [28], and also can be regulated by IL-17 to drive neutrophil migration and mediate tissue injury [42-44]. Moreover, the high expression of IFN-γ could inhibit IL-10 production [45], consistent with our results showing that IL-10 levels were decreased in jejunum and ileum. In addition, IFN-γ can disrupt the colonic epithelial barrier and increase intestinal tight junction permeability [24-26]. Many mechanisms have been proposed for this phenomenon, including dysbiosis and elimination of Paneth cells [46]. Proliferation of intestinal epithelial cells is inhibited through suppression of β-catenin/T cell factor signaling [47], and the AMPK signaling pathway is activated by phosphorylation [48]. IL-17,which plays a protective role in infections, exhibits its inflammatory effects by activating NF-κB, MAPKs and C/EBP cascades to induce the production of multiple pro-inflammatory molecules and subsequent activation of macrophages and neutrophils [49]. It also has a regulatory function limiting the accumulation and activity of neutrophils by attenuating the anti-apoptotic effect of inflammatory cytokines during the inflammatory process [50]. In our PI-IBS model, by 56 days PI, *T. spiralis* was completely absent from the intestinal mucosa but IL-17 still persisted, suggesting that increased IL-17 in the duodenum and ileum may be vital for maintaining intestinal low-grade inflammation. First, the IL-23/IL-17 signal pathway (which regulates IL-12/IFN-γ, and drives neutrophil migration to mediate inflammation injury) has been well described [42-44]. Second, uncontrolled IL-17 responses can augment production of inflammatory factors including IL-1, IL-6, IL-8, TNF-α, GM-CSF, and MIP**-**2 [44,51]. These cytokines were reported to be increased in the peripheral blood and intestinal mucosa of PI-IBS patients [20], which would alter the gut physiology and immunity of the host, causing clinical symptoms [20,36].

IL-10, as a classical anti-inflammatory cytokine, decreases the inflammatory reaction through a number of mechanisms. It can diminish the production of inflammatory mediators including IL-1β and IFN-γ in T cells and activate macrophages [52-54]. It also can reduce the expression of major histocompatibisblity complex class II, co-stimulating, and adhesion molecules on the surface of antigen-presenting cells [55,56]. Importantly, it can also suppress the development of mast cells [57]. In our study, IL-10 levels were decreased in the jejunum, ileum and colon of PI-IBS model mice. Because of the decreased IL-10, on one hand pro-inflammatory cytokines were much more highly expressed, resulting in intestinal low-grade inflammation persisting. On the other hand, the antigen-presenting cells maintained their function, contributing to the adaptive immune response. Furthermore, hyperplasia of mast cells may be related to the decreased IL-10 levels [57], and they can also alter homeostatic intestinal epithelial migration and barrier function [57,58].

In the present study, the expression levels of IL-1β were not significantly different between the two groups. Further research is required to explore whether IL-1β levels were suppressed by anti-inflammatory cytokines other than IL-10. Although changes of the four cytokines that we measured were not found in all intestinal segments, it is reasonable to deduce that the physiology and immunity of the host may be impaired by other cytokines [47,59].

## Conclusions

This study indicates that visceral hypersensitivity persists after signs of chronic intestinal inflammation have disappeared. The observed long-term colonic hypersensitivity appears to be at least partially mediated by cytokines, because there is an imbalance of cytokines. The changes of cytokines in different intestinal segments may alter the imbalance between pro- and anti-inflammatory reactions within the gut. This suggests that the local low-grade inflammation and immune activation that are an important component of the pathophysiology of PI-IBS may be partly induced and maintained by cytokines. This may also explain why PI-IBS patients present with abdominal distension, abdominal pain and other clinical symptoms.

## Abbreviations

PI-IBS: Post-infection irritable bowel syndrome
IL-1β: Interleukin-1-beta
IL-10: Interleukin-10
IL-17: Interleukin-17
IFN-γ: Interferon-gamma
PI: Post-infection
AWR: Abdominal withdrawal reflex
